# Shedding light on quality of care: a study protocol for a randomized trial evaluating the impact of the Solar Suitcase in rural health facilities on maternal and newborn care quality in Uganda

**DOI:** 10.1186/s12884-019-2453-x

**Published:** 2019-08-22

**Authors:** Slawa Rokicki, Brian Mwesigwa, Laura Schmucker, Jessica L. Cohen

**Affiliations:** 1 Geary Institute for Public Policy, University College Dublin, Dublin, Ireland; 2 Department of Health Behavior, Society & Policy, Rutgers School of Public Health, Piscataway, USA; 3 Innovations for Poverty Action, Kampala, Uganda; 4 Department of Global Health and Population, Harvard T.H. Chan School of Public Health, Boston, USA

**Keywords:** Quality of care, Maternal health, Newborn health, Electricity, Solar energy, Infrastructure, Stepped wedge cluster randomized trial, Uganda

## Abstract

**Background:**

Continued progress in reducing maternal and newborn morbidity and mortality in low-income countries requires a renewed focus on quality of delivery care. Reliable electricity and lighting is a cornerstone of a well-equipped health system, but most primary maternity care facilities in sub-Saharan Africa are either not connected to the electrical grid or suffer frequent blackouts. Lack of reliable electricity and light in maternity facilities may contribute to poor quality of both routine and emergency obstetric and newborn care, by hindering infection control, increasing delays in providing care, and reducing health worker morale. The “Solar Suitcase” is a solar electric system designed specifically for maternity care facilities in low-resource environments. The purpose of this trial is to evaluate the impact of the Solar Suitcase on reliability of light, quality of obstetric and newborn care, and health worker satisfaction.

**Methods:**

We are conducting a study with 30 maternity care facilities in rural Uganda that lack access to a reliable, bright light source. The study is a stepped wedge cluster randomized controlled trial. Study facilities are identified according to predefined eligibility criteria, and randomized by blocking on baseline covariates. The intervention is a “Solar Suitcase”, a complete solar electric system that provides essential lighting and power for charging phones and small medical devices. The primary outcomes are the reliability and quality of light during intrapartum care, the process quality of obstetric and newborn care, and health worker satisfaction. Outcomes will be assessed via direct clinical observation by trained enumerators (estimated *n* = 1980 birth observations), as well as interviews with health workers and facility managers. Lighting and blackouts will be captured through direct observation and via light sensors installed in facilities.

**Discussion:**

A key feature of a high quality health system is appropriate infrastructure, including reliable, bright lighting and electricity. Rigorous evidence on the role of a reliable light source in maternal and newborn care is needed to accelerate the “electrification” of maternity facilities across sub-Saharan Africa. This study will be the first to rigorously assess the extent to which reliable light is an important driver of the quality of care experienced by women and newborns.

**Trial registration:**

ClinicalTrials.gov: NCT03589625 (July 18, 2018); socialscienceregistry.org: AEARCTR-0003078 (dated June 16, 2018).

**Electronic supplementary material:**

The online version of this article (10.1186/s12884-019-2453-x) contains supplementary material, which is available to authorized users.

## Background

Every year in sub-Saharan Africa (SSA), 1.2 million women and newborns die in delivery or shortly thereafter [1, 2]. Maternal health policies and programs aimed at reducing home births over the past decade have been very successful—with rates of facility-based delivery increasing dramatically—but increases in facility births have often not led to meaningful improvements in maternal and newborn mortality and morbidity [3–7]. A common explanation for this disconnect is the poor quality of care provided by many maternity facilities, including lack of highly-skilled personnel, insufficient supplies and equipment, and poor infrastructure such as reliable access to light and electricity [8]. A recent study assessing the quality of basic maternal care functions across five African countries found that nearly 90% of primary maternity care facilities and 34% of secondary maternity care facilities lack provision of electricity [8]. A study across eight SSA countries found that among facilities with access to electricity, on average only 28% reported reliable access [9]. 

Lack of light and electricity is a major challenge to providing adequate delivery care at night: qualitative interviews from Kenya found that health workers rely on torches, kerosene lamps, and lights from mobile phones to conduct procedures [10]. These conditions can make even the most routine deliveries unsafe—e.g. hindering the ability to monitor the progression of labor or follow infection control protocols—as well as critically challenge providers’ ability to manage emergency complications such as post-partum hemorrhage and newborn asphyxia. Health workers may delay life-saving care, for example, when they cannot identify the site of tears due to insufficient light, or when there is only one light for both mother and infant [11]. A number of studies have found that delays in receiving obstetric care at the health facility, known as the ‘third delay’ in the standard three delays model [12], is associated with poor maternal and infant outcomes [13–16]. Finally, lack of sufficient light may also increase stress on the part of the health worker, which may lead to errors in provision of care.

Although reliable electricity appears foundational to safe delivery, evidence of the impact of electrification on quality of care or health outcomes is sparse, particularly causal estimates [17]. Several cross-sectional studies have examined the association between reliability of light and electricity in health facilities with quality of care and mortality outcomes [8, 18–20]. However, cross-sectional studies may be biased by confounding factors that affect both access to electricity and health outcomes, as well as possible reverse causality, for example, if higher quality health workers advocate for better electricity.

Continued progress toward Sustainable Development Goals in both health and access to clear modern energy will require that all women deliver in facilities with reliable electricity [21]. However, expanding large power grid systems to African maternity facilities would increase greenhouse gas emissions and strain already failing infrastructure, while relying on polluting diesel generators is unsustainable in the face of volatile petroleum prices [19]. Investment in solar energy systems for health care facilities could effectively hasten the transition away from fossil fuels, while promoting health and development [22]. The “Solar Suitcase” is a complete solar electric system that provides essential lighting and power for charging phones and small medical devices. Solar suitcases have been designed specifically for maternal health facilities in low-resource environments [11].

We are conducting a study with maternity care facilities in rural Uganda that lack access to a reliable, bright light source. The study is a stepped wedge cluster randomized controlled trial evaluating the impact of the “Solar Suitcase” on the reliability and quality of light during intrapartum care, the process quality of obstetric and newborn care, and health worker satisfaction.

## Methods

### Study context

Uganda has a population of about 34.6 million people, with 48% below the age of 15, and an estimated 79% living in rural areas [23]. About 29% of households in Uganda have access to electricity, though only 18% of rural households do [24]. The total fertility rate in 2017, at 5.5 children per woman, was higher than the average in low income countries (4.6 children) [25]. Nearly all women (97%) receive antenatal care from a skilled provider and 73% of births are delivered in a health facility [24]. The maternal mortality ratio for Uganda is 336 deaths per 100,000 live births for the period of 2009–2016. The perinatal mortality rate—defined as stillbirths after seven completed months gestation and deaths within the seven days after birth—is an indicator directly linked to the quality of antenatal and intrapartum care. In Uganda, the perinatal mortality rate is 38 deaths per 1000 pregnancies for the period of 2011–2016 [24], and this rate has remained stagnant since 2006 [26]. A study on delays in newborn care in Uganda found that delays in receiving quality care at the health facility was the second major contributor to newborn deaths, and that facilities were ill-equipped to care for newborns [27].

Uganda’s health system is divided into public and private sectors [28]. The public sector includes national and regional hospitals and a District Health system composed of a tiered system of health centers (HCs). The health center tiers include: (1) HC I’s, community-run health volunteers, (2) HC IIs, first level of interface between the formal health sector and the community providing outpatient services and some maternity services where necessary, (3) HC III’s, offering outpatient and inpatient services, including uncomplicated maternity care services, and (4) HC IV’s, offering provision of emergency medical, surgical, and obstetrical care (such as caesarean section) [28, 29].

Recent, high quality data on health facility access to reliable light and electricity in Uganda is sparse. Research has generally found that while most HC IV’s have access to the central supply electricity grid, most HC IIs and IIIs do not, and often rely on kerosene lamps for much of their lighting needs [30–32]. While kerosene lamps can provide ambient lighting, it is not sufficient for providing the focused, bright light that many elements of maternity care requires [10]. In addition, even among facilities connected to the grid, 74% had frequent interruptions lasting more than two hours a day [33].

### Intervention

**Fig. 1:**
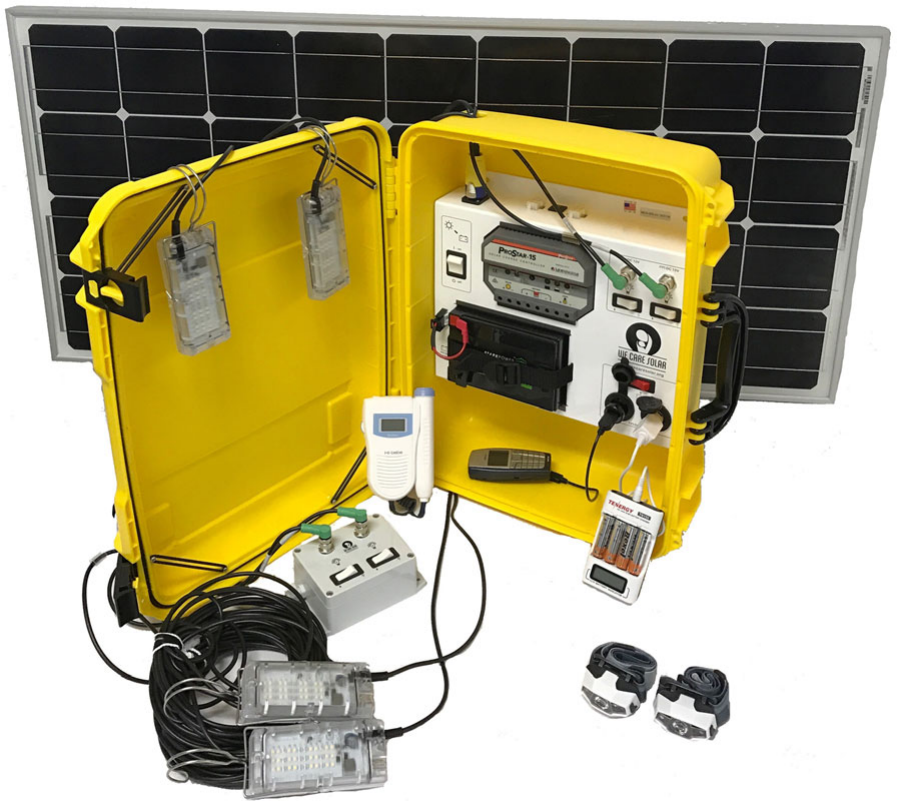
Image of The We Care Solar Suitcase® and component parts. Notes: Copyright by We Care Solar. Written permission was given by We Care Solar to reproduce this image

The intervention is a “Solar Suitcase” manufactured by the non-governmental organization We Care Solar. The We Care Solar Suitcase® is a complete solar electric system that provides essential medical lighting and power for charging phones and small medical devices (Fig. 1). The system contains a photovoltaic solar panel installed on the roof of a health facility; a 12 V lithium ferrous phosphate battery; high-efficiency, moveable light-emitting diode (LED) lights for maternity rooms; and two rechargeable LED headlamps. In addition, it contains a fetal Doppler with rechargeable batteries, two 12 V DC accessory sockets, two USB ports for charging cell phones, and a AA/AAA battery charger. Because the Solar Suitcase includes several components, the evaluation will test the overall impact of all components on outcomes and it will not be possible to determine the impact of any individual component.

Installations will be done by a local solar contracting firm based in Uganda. Depending on the facility, each installation takes 3–6 h. One Solar Suitcase will be installed in each facility, with 2–4 overhead LED lights for each delivery room, depending on its size. To ensure consistent and appropriate use of the installed Suitcase, installers will teach health workers how to use and maintain the Solar Suitcase and all accessories on the day of installation and in subsequent check-ins, which will be done in-person or over the phone as needed. The contractor will also follow up with maintenance requests. Health facilities will not incur any costs during the study for installation, operation, or maintenance of the Solar Suitcase.

### Trial design

The study is designed as a stepped wedge cluster-randomized controlled trial. The intervention is delivered at the cluster (i.e. facility) level and the primary outcome is measured at the birth event level (i.e. mother and infant). Facilities are randomized into one of two groups of 15 facilities. The stepped wedge design ensures that both groups of facilities eventually receive the intervention, but the implementation is staggered in a random fashion allowing a rigorous evaluation to take place. All facilities are observed before and after receiving the intervention. Regardless of study findings, at the end of the study all facilities will keep the Solar Suitcase and will be provided with contact information in case of needed repairs.

**Fig. 2:**
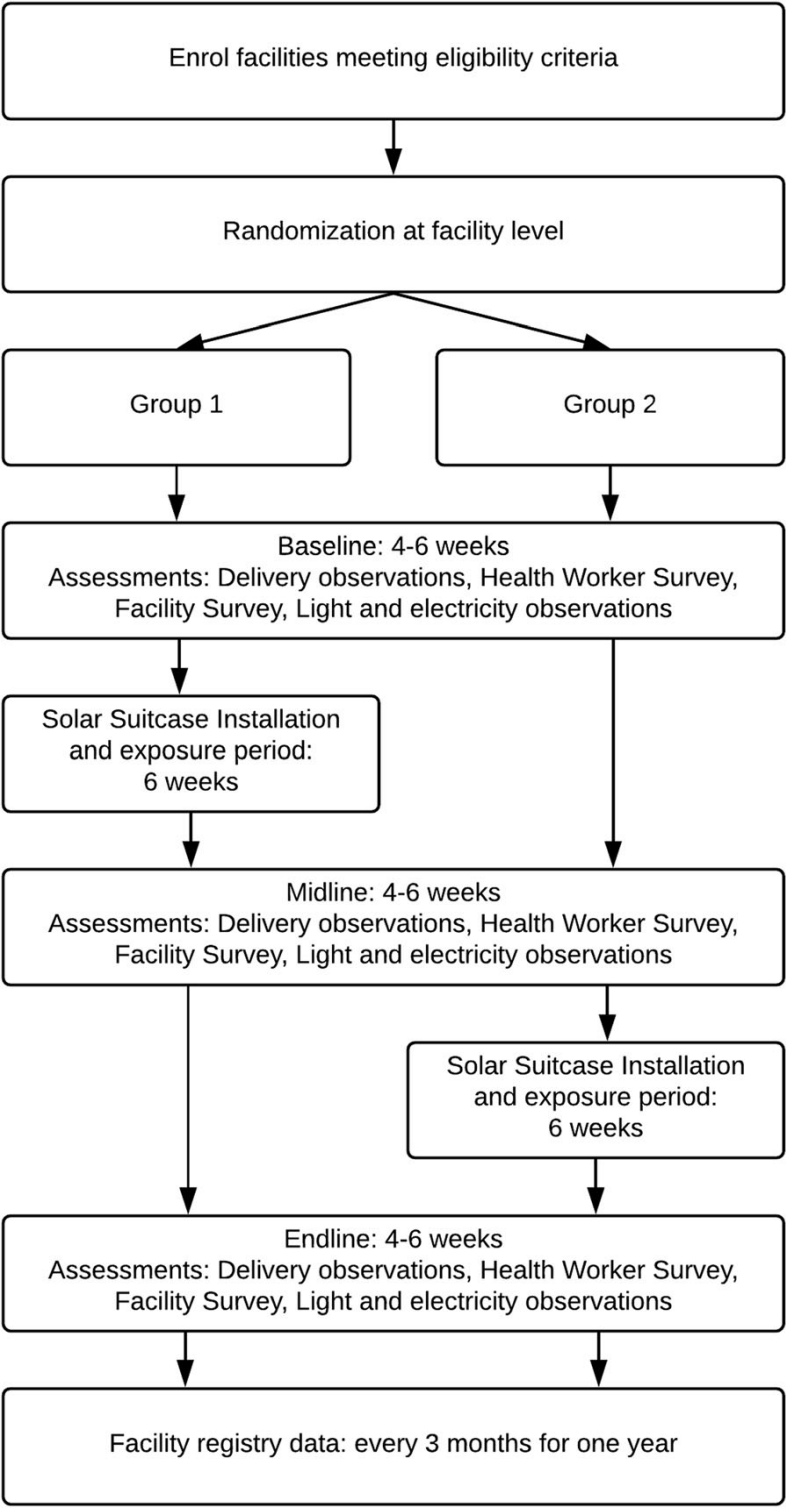
Flowchart of enrolment, randomization, and assessments during study period

The study timeline (Fig. 2) includes a baseline (pre-intervention) observation period of about 4–6 weeks, followed by installation of the Solar Suitcase in the first group of facilities. After installation, there is an exposure period of about 6 weeks so that facility staff can become comfortable with the Solar Suitcase. Following this period, there is a midline observation period of 4–6 weeks, which serves as a post-intervention observation for group 1 and a second pre-intervention observation for group 2. Finally, installation will occur in the second group of facilities, followed by another 6 week exposure period and then an endline observation of 4–6 weeks, serving as a second post-intervention observation for both groups.

Study facilities are identified according to the following predefined eligibility criteria: i) facility is level II, III, or IV; ii) facility-reported overhead light is unreliable (zero or one functioning sources of overhead light in the maternity ward, which is interrupted at least a few times a week); iii) facility does not automatically refer out when there is no light; iv) facility is open 24 h a day; and v) medical officer in-charge (MOIC) at the facility is willing to participate. Enumerators will collect data at each facility for a trial period to verify study eligibility (i.e. to verify light reliability and source).

The facilities will be chosen from districts in Central, Eastern, and Western regions of Uganda. Approval and support for working in these districts was granted by the Ministry of Health (MOH) of Uganda as well as the District Health Officers and Resident District Commissioners.

For patients delivering at participating facilities, inclusion criteria includes consenting pregnant women aged 16 and older who are admitted for labor and delivery. Exclusion criteria includes pregnant women presenting for conditions other than normal labor and delivery, women who are immediately transferred to another hospital, women who deliver outside of the maternity ward, or women for whom the health worker objects to the observation.

Enumerators will have a minimum of a diploma or certificate in Comprehensive Nursing or Midwifery from an accredited Uganda National Council of Higher Education. Enumerators will be trained for approximately 2 weeks on research protocol, data collection methods, and human subjects research.

### Randomization

Facilities will be randomized to receive the intervention at the first step or the second step. Data collection will occur in three staggered waves. Randomization will be blocked by wave and by a measure of average adequate light during baseline (above or below the median for all facilities), and balanced on a 20-item quality of care index measured at baseline and average facility volume over the past three months. The algorithm will re-randomize until the balance statistic (Wilks’ lambda on a manova) exceeds 0.2. The allocation sequence will be generated by the study investigators using Stata version 15. Due to the nature of the intervention, neither participants nor researchers can be blinded to allocation.

### Data collection

The following six types of data will be collected in this study.

#### Delivery observation checklist

Quality of care will be assessed via direct clinical observation of deliveries by enumerators. Enumerators will complete a checklist for each delivery capturing the elements of care and timeliness of care provided to each eligible, consented patient. The checklist tool is adapted from the Maternal and Child Health Integrated Program Quality of Care Surveys and Short Observational Index [34]. Additional checklists are also used for cases of multiple birth, resuscitation of newborn with asphyxia, and observation of post-partum hemorrhage.

#### Health worker survey

Interviews will be conducted with all consenting medical staff who may be involved in labor and delivery. Information will be collected on basic sociodemographic characteristics, background and training, perception of electricity reliability in the facility, hours and duties, satisfaction and personal drive, and knowledge of maternal and newborn care.

#### Facility survey

The facility survey includes a general facility assessment and the recording of facility registry data and will be conducted with the MOIC and/or the Head Midwife (maternal ward lead). This assessment will contain information on the facility infrastructure, staffing, services, fees, drug/supplies availability, and reported performance of routine and emergency signal functions.

Recording of facility registry data will consist of collecting data from the MOH Health Management Information System (HMIS), including number of vaginal deliveries, number of cesarean deliveries, number of nighttime deliveries, number of referrals, and number of ANC visits.

#### Light and electricity observation

Information on availability and quality of light will be recorded in two ways. First, enumerators will observe and record sources of light, brightness of light, and timing in changes to sources of light on a data collection form. Second, light sensors will be installed in delivery rooms of health facilities, which will collect light voltage data for the duration of the study. For facilities connected to the grid, monitors will also be installed to detect whether the grid power is on or off. Data collection on availability and quality of light will be conducted during both daytime and nighttime hours.

#### Facility volume

For one year after completion of birth observations, HMIS facility data on number of mothers presenting for delivery, number of nighttime births, number of live births, number of newborn deaths, number of newborn resuscitations, and number of ANC visits will be collected at quarterly visits to facilities.

#### Qualitative observations

Enumerators will record qualitative observations of deliveries, including commentary on how light and electricity may have influenced the care provided, as well as contextual information about facility supplies, staffing, operations, and use of the Solar Suitcase components that may provide insight into interpretation of quantitative data.

### Outcomes

The evaluation of this study will include primary and secondary outcomes, as well as process measures and qualitative assessments of impact. Our study has more pre-specified outcomes than is typical for a randomized controlled trial because the important aspects of light (the brightness, the light source, or both) and the precise ways in which reliable electricity may affect quality of care are difficult to predict ex-ante. We thus chose several measures of light and several indices that capture various elements of quality of care and delays in providing care throughout labor, delivery and the immediate post-partum period.

The primary outcomes include measures of adequate light, quality of care, and health worker satisfaction (Table 1). Measures of brightness are assessed by enumerators with standardized training as to what level of light defines each term. These outcomes will be evaluated during both daytime and nighttime hours, as facilities may experience dim conditions during the day and the Solar Suitcase provides a bright moveable light that can be used for difficult, detail-oriented work such as suturing.

**Table 1:** Primary outcomes

Category	Outcome
Light	1. Average brightness of room during labor and delivery as measured by observer recorded questionnaire (range: 1 “pitch black” – 4 “very bright”) 2. Satisfactory light source^a^ used for entire delivery as measured by observer recorded questionnaire (%). 3. Adequate light^b^ for duration of delivery as measured by observer recorded questionnaire (%).
Quality of care	1. 20-item quality of maternal care index of essential actions to be performed by provider during labor and delivery as measured by observer recorded questionnaire (%) 2. 37-item quality of maternal care index of essential actions to be performed by provider during labor and delivery as measured by observer recorded questionnaire (%) 3. 6-item delays in care index as measured by observer recorded questionnaire
Health Worker Satisfaction	1. Satisfaction with light & electricity^c^ as measured by health worker survey (range 1 “strongly disagree” – 5 “strongly agree”) 2. Overall job satisfaction index^d^ as measured by health worker survey (range 1 “strongly disagree” – 5 “strongly agree”)

Notes. ^a^“Satisfactory” defined as observation that occurs during the day or observation that occurs during night and uses grid electricity, solar power, or functional generator (rather than kerosene lamp, candle, or torch)

^b^“Adequate” light defined as light from a satisfactory source and is recorded by enumerator as “very bright” or “somewhat bright” (rather than “dim” or “pitch black”)

^c^Satisfaction defined as agree/strongly agree with both statements: "I am satisfied with the availability and brightness of light in this facility" and "I am satisfied with the availability of electricity in this facility"

^d^Overall satisfaction defined as average score of following items: "i) These days, I feel motivated to work as hard as I can. ii) Overall, I am satisfied with my job. iii) Overall, the morale level at my department is good iv) I plan on staying at this position for the next year"

The 20-item provision of quality of care index is a validated measure developed for and evaluated in low- and middle-income settings [35]. It is composed of 20 indicators representing key dimensions of the quality of the process of intrapartum and immediate postpartum care in facility deliveries (Table 2). The index captures elements of quality including initial patient assessment, labor monitoring and delivery, and the immediate post-partum period (one hour after delivery). Research applying the index in three countries in sub-Saharan Africa found that it effectively discriminates between poorly and well-performed deliveries [35]. The 37-item index includes all 20 items plus an additional 17 items adapted from the Maternal and Child Health Integrated Program tool [34] (Additional file 1: Table A1). Both indices are constructed as the proportion of items performed per delivery and thus range from 0 to 1.

**Table 2:** Items composing the 20-item quality of maternal care index, Tripathi (2015)

Initial client assessment and examination	
1. Checks woman’s HIV status (checks chart or asks woman) and/or offers woman HIV test	
2. Asks whether woman has experienced headaches or blurred vision	
3. Asks whether woman has experienced vaginal bleeding	
4. Takes blood pressure	
5. Takes pulse	
6. Washes his/her hands before any examination	
7. Wears high-level disinfected or sterile gloves for vaginal examination	
First stage of labor	
8. At least once, explains what will happen in labor to the woman and/or her support person	
9. Prepares uterotonic drug to use for AMTSL	
10. Uses partograph (during labor)	
11. Self-inflating ventilation bag (500 mL) and face masks (size 0 and size 1) are laid out and ready for use for neonatal resuscitation	
Second and third stage of labor	
12. Correctly administers uterotonic (timing, dose, route)	
13. Assesses completeness of placenta and membranes	
14. Assesses for perineal and vaginal lacerations	
Immediate newborn and postpartum care	
15. Immediately dries baby with towel	
16. Places newborn on mother’s abdomen skin-to-skin	
17. Ties or clamps cord when pulsations stop, or by 2–3 min after birth (not immediately after birth)	
18. Takes mother’s vital signs	
19. Palpates uterus	
20. Assists mother to initiate breastfeeding within one hour	

Notes: AMTSL = active management of the third stage of labor. Each indicator is scored as binary for health worker performed (1) or did not perform (0). Index score for each delivery is proportion of items performed. Reference for index is [35]

The 6-item delays in care index measures the average of 6 measures of delays in time to provision of care (measured in minutes), and includes time between: facility arrival and first contact with health care worker, facility arrival and first examination, delivery and provision of uterotonic, delivery and assessment of perineal and vaginal lacerations, delivery and drying baby with towel, and delivery and initiation of breastfeeding.

Finally, we include two measures of health worker satisfaction: (1) satisfaction with the availability and brightness of light and electricity (2-item index), and (2) overall job satisfaction including motivation, job satisfaction, morale, and plans to leave (4-item index). Items composing these indices and measurements are shown in Additional file 1: Table A2. Indices were adapted and extended from the World Bank Impact Evaluation Toolkit for Results Based Financing in Health [36].

Secondary outcomes are shown in Table A3 of Additional file 1 and include the individual components of the quality indices as well as other outcomes that may be affected by the provision of a reliable light source such as referrals out of the facility, patient volumes, APGAR scores, and suturing.

#### Power analysis and sample size

Our sample will include 30 facilities. We expect to observe about 1980 patients (22 per facility at each of the three data collection points), and about 90 health workers (an average of 3 per facility). We used data collected from pilot facilities (that are not included in the study sample) to construct sample size and power estimates prior to study launch. We then updated these calculations with data collected at baseline with study facilities since this is likely to be a more accurate prediction of statistical power for study facilities. (Calculated effect sizes do not differ meaningfully if pilot data or baseline data is used.) We use this data to calculate means of outcome variables and the intraclass correlation (ICC). These power calculations use the full stepped wedge design (using the steppedwedge function in Stata v15), for 22 birth events per facility, 3 health workers per facility, 2 steps, 80% power and 0.05 alpha.

We discuss here the minimum detectable effect sizes for one outcome within each domain of light, quality, and health worker satisfaction. We estimate that 61% of deliveries at baseline will be conducted with an adequate light source, with an intra-cluster correlation (ICC) of 0.20. Our minimum detectable effect size for this outcome is an increase of 13 percentage points. We estimate that health workers will be performing 44% of the essential quality of care items in the 20-item quality of care index (i.e. a score of .44) with an ICC of 0.4. Our minimum detectable effect size for this outcome is an increase of 11 percentage points (i.e. an increase to .54), representing an increase in the performance of about 2 items. We estimate that health worker satisfaction with light and electricity is a 2 (out of a 1–5 range) with an ICC of 0.3. Our minimum detectable effect size for this outcome is an increase of 0.62.

### Ethical considerations

Oversight of research in Uganda involving human subjects requires two levels of approval: (1) approval is required at the organizational level by Mildmay Uganda Research Ethics Committee and (2) approval is required at the national level by the Uganda National Council for Science and Technology in collaboration with the Uganda National Health Research Organization. Both levels of approval were obtained, alongside full review by the Harvard School of Public Health Institutional Review Board (IRB) and exemption from review by University College Dublin.

#### Consent procedures

Consent to participate is voluntary and participants, including facilities, health workers, and patients, can withdraw at any time. Written informed consent for all activities pertaining to the study will be obtained from the MOIC and all health workers who may be involved in labor and delivery prior to conducting observations and interviews. Written informed consent from patients will be obtained prior to or just after admission. Consent forms for patients will be available in seven local languages, with enumerators posted to facilities in areas where they speak the local language. If either the health worker or patient does not consent or objects to the observation after providing consent, the delivery observation will not occur. In such a case where the patient opts out, the care provided to the patient (and the baby) during the admission and after discharge will not be any different from what she, and the baby, would have received as a study participant.

#### Data quality and privacy

Data collection will take place using both paper surveys and electronically on tablets. Double data entry will be conducted and discrepancies will be reconciled. Logic checks for electronic entry will also be put into place to ensure linearity of timestamps and prevent contradictory data.

Every birth event will be allotted a unique code. Observation data collection forms will be anonymous and no identifiers will be listed on these forms. No personal identifying information of women delivering will be collected on the paper or electronic questionnaires. The names of health care workers will be collected in order to link them to an identification number. The names and corresponding identification numbers will be kept securely in a locked cabinet. Results presented in reports and publications will always be aggregated in such a way that identification of facilities, health workers and patients is not possible. Data from tablets will be uploaded to an encrypted data storage cloud system managed by the research management team in Uganda and any data stored locally will be stored in encrypted folders.

#### Monitoring

The PIs and research management team will have bi-weekly calls to discuss study progress. These meetings will be held to monitor data collection and discuss any adverse events, protocol violations, or protocol modifications. Any issues will be reported to the PIs within 24 h. In-person oversight of data collection from the management team will be ongoing throughout the study and data quality checks will take place daily. Additionally, there will be biweekly in-person group feedback sessions for enumeration staff to share challenges of conducting observations and discuss ways to overcome those challenges.

There is no Data and Safety Monitoring Board, interim analyses, or stopping guidelines because there is no greater than minimal risk for participants, as deemed by the IRB.

### Data analysis and dissemination

Analysis will be by intention-to-treat, with adjustments made for multiple hypothesis testing. Let *Y*_*ijt*_ be the outcome of interest for birth event *i* in facility *j* at time *t*, with *t* measured as the time-step. *D*_*jt*_ is a binary variable for whether the Solar Suitcase had been installed at facility *j* at time *t*.

The specification for the linear predictor representation is shown below:

$$ {Y}_{ijt}={A}_j+{B}_t+c{X}_{jt}+\beta {D}_{jt}+{\epsilon}_{ijt} $$

where *A*_*j*_ are facility fixed effects, *B*_*t*_ are calendar month fixed effects, *X*_*jt*_ is a matrix of facility-level controls, and *ϵ*_*ijt*_ is the error term. The estimated impact of installation of the Solar Suitcase is $\hat{\beta}$. We will also analyze the impact of the Solar Suitcase at each step separately, i.e. *D*_*jt*_ will be defined as a 3-level indicator (baseline, 1st follow-up post installation, 2nd follow-up post installation). In primary analyses, we will adjust for length of time spent observing at a facility in *X*_*jt*_. In secondary analyses, we will adjust for additional baseline covariates to account for any baseline imbalances. Standard errors will be adjusted for clustering at the facility level and will be compared to those obtained from a wild cluster bootstrap. In a sensitivity analysis, we will conduct a multilevel regression with facility random effects instead of fixed effects, adjusting for randomization blocking variables and a variable accounting for length of time spent observing at a facility.

For linear outcomes, analysis will consist of linear regression models. For count outcomes, analysis will consist of Poisson or negative binomial regression models. For binary outcomes, analysis will consist of logistic regression models. For the outcome of health worker satisfaction, the specification will be at the health worker level, rather than the birth-event level.

We will conduct analysis using all of the pooled data and using data of deliveries occurring in the night-time only. We will also conduct a heterogeneity analysis, exploring whether bigger changes in light availability are associated with bigger improvements in quality of care.

The study investigators will have access to the final trial dataset and interim datasets before the intervention is implemented. The study results will be disseminated to internal and external collaborators, funders, scientific media, and policymakers at the MoH. After manuscript publication, fully de-identified data may be shared with researchers upon request.

## Discussion

Continued progress in reducing maternal and newborn severe morbidity and mortality requires a renewed focus on quality of care. The maternal health community has called for a “quality revolution” [37], urging policy-makers and researchers to focus on improving access to safe, effective and respectful delivery care. A key feature of a high quality health system is appropriate infrastructure. Reliable, bright lighting and electricity is a cornerstone of a well-equipped maternity facility. This study will be the first to rigorously assess the impact of providing a reliable, high quality light source on the quality of delivery care provided to women and newborns.

Beyond assessing the overall impact of reliable light and electricity on quality of care, this study will help determine precisely which aspects of care are most influenced by light availability in this context. This study will also be among the first to document critical delays in the provision of maternal and newborn care using clinical observation, and will assess the extent to which care during nighttime and blackouts suffers from critical delays in patient intake and assessment and in immediate post-partum care. Finally, our study can provide insight into the role of light in non-technical aspects of delivery care, including providers’ feelings of work satisfaction and safety, and observations of respectful care. While a number of previous studies have documented variation in the quality of maternal care across sub-Saharan Africa [5, 7, 8, 38], very little is known about the reasons for low-quality care. Our study addresses the extent to which reliable light is an important driver of the quality of care experienced by women and newborns.

Our study has a number of strengths. First, it uses a randomized design in order to rigorously determine impact. Second, it is a stepped-wedge randomized trial, so that no facilities are denied access to the intervention. Third, we use direct clinical observation of quality of care and time stamping, rather than relying on retrospective self-reports or documentation, which can suffer from recall bias, desirability bias, and other forms of measurement error. Fourth, we collect data on conditions of light both objectively (via light sensors) and subjectively (via enumerator report), which we can match with the care provided at the time. Fourth, our data collection tools are detailed enough to allow us to trace out the primary mechanisms by which reliable light and electricity may influence quality of care.

This study also has several limitations. First, the number of clusters (facilities) included in our sample is somewhat small for a cluster randomized trial and the intra-cluster correlation in some of our primary outcomes is quite high. We will need to adjust standard errors to account for the number of clusters and for the degree of intra-cluster correlation and this weakens our power to detect very modest effect sizes of the intervention on some outcomes. Second, we are exploring the impact of light on a number of different dimensions of care quality and, although these outcomes are pre-specified, we will need to adjust for multiple hypothesis testing, which could reduce statistical power. Third, the Solar Suitcase intervention includes several different components, including task lights, headlamps, and a fetal Doppler; however, our analysis will not be able to distinguish which components are most useful or whether the components are more effective as a whole than separately. However, we will collect qualitative data that may provide some information regarding uptake of each component. Fourth, our results are limited to rural maternity facilities in Uganda and may not be generalizable to higher level facilities and hospitals or to maternal care outside of Uganda. Finally, it is possible that the presence of an observer influences health care providers’ behavior (known as the Hawthorne effect) so that our data would not accurately capture the quality of care that would be provided without an observer. Enumerators will be trained to be as unobtrusive as possible during observations by avoiding any communication or interaction with health workers or patients during observation (outside of obtaining consent to participate), and by situating themselves in a location that allows them to see the health worker’s actions but is as unimposing as possible. Because enumerators conduct multiple observations over several weeks in each facility, this may help normalize their presence over time. However, this limitation remains.

In low-income countries like Uganda, where many health centers are either not connected to the electrical grid or suffer frequent blackouts, many deliveries occur x lamps. In addition to straining health workers’ ability to provide adequate care, the absence of reliable, high quality light can make patients and health workers feel unsafe. It can also cause health workers to refer away women who come to deliver at night, potentially leading to dangerous delays while traveling to another facility, and may deter women from delivering at facilities in the first place [39]. We expect our study to illuminate some of the ways that the provision of a reliable, high quality lighting source can improve the quality of care women and newborns receive. This evidence can in turn help build the case for prioritizing investment in reliable electricity as part of a high quality health system. On the other hand, it is possible that even when a reliable light source is supplied, overall quality of care does not meaningfully improve. For example, improved lighting may not influence quality of care if health worker training or incentives are insufficient. While our study is not designed specifically to tease out these interactions, this is an important priority for future research on how to build high quality health systems that protect and respect mothers and newborns.

## Trial status

The trial is ongoing. It is currently conducting data collection.

## Additional file

Additional file 1: **Table A1**. Additional 17 items composing 37-item quality of maternal care index, as adapted from MCHIP (2013). **Table A2**. Items composing satisfaction indices. **Table A3**. Secondary outcomes. (DOCX 19 kb)

## Data Availability

Data sharing is not applicable to this article as no datasets were generated or analyzed during the current study.

## Abbreviations

HC: Health center
HMIS: Health Management Information System
ICC: Intraclass correlation
IRB: Institutional Review Board
LED: Light-emitting diode
MoH: Ministry of Health
MOIC: Medical Officer In-Charge
SSA: Sub-Saharan Africa

## References

[1] Alkema L, Chou D, Hogan D, Zhang S, Moller A-B, Gemmill A (2016). Global, regional, and national levels and trends in maternal mortality between 1990 and 2015, with scenario-based projections to 2030: a systematic analysis by the UN maternal mortality estimation inter-agency group. Lancet.

[2] Lawn JE, Blencowe H, Oza S, You D, Lee AC, Waiswa P (2014). Every newborn: progress, priorities, and potential beyond survival. Lancet.

[3] Murray SF, Hunter BM, Bisht R, Ensor T, Bick D (2014). Effects of demand-side financing on utilisation, experiences and outcomes of maternity care in low- and middle-income countries: a systematic review. BMC Pregnancy Childbirth..

[4] Chaturvedi S, Randive B, Diwan V, Costa AD (2014). Quality of obstetric referral Services in India’s JSY cash transfer Programme for institutional births: a study from Madhya Pradesh Province. PLoS One.

[5] Austin A, Langer A, Salam RA, Lassi ZS, Das JK, Bhutta ZA (2014). Approaches to improve the quality of maternal and newborn health care: an overview of the evidence. Reprod Health.

[6] Souza JP, Gülmezoglu AM, Vogel J, Carroli G, Lumbiganon P, Qureshi Z (2013). Moving beyond essential interventions for reduction of maternal mortality (the WHO multicountry survey on maternal and newborn health): a cross-sectional study. Lancet.

[7] Miller S, Abalos E, Chamillard M, Ciapponi A, Colaci D, Comandé D (2016). Beyond too little, too late and too much, too soon: a pathway towards evidence-based, respectful maternity care worldwide. Lancet.

[8] Kruk ME, Leslie HH, Verguet S, Mbaruku GM, Adanu RM, Langer A (2016). Quality of basic maternal care functions in health facilities of five African countries: an analysis of national health system surveys. Lancet Glob Health.

[9] Adair-Rohani H, Zukor K, Bonjour S, Wilburn S, Kuesel AC, Hebert R, Fletcher ER (2013). Limited electricity access in health facilities of sub-Saharan Africa: a systematic review of data on electricity access, sources, and reliability. Glob Health Sci Pract.

[10] Essendi H, Johnson FA, Madise N, Matthews Z, Falkingham J, Bahaj AS (2015). Infrastructural challenges to better health in maternity facilities in rural Kenya: community and healthworker perceptions. Reprod Health.

[11] Humphreys G (2014). Harnessing Africa’s untapped solar energy potential for health. World Health Organ Bull World Health Organ Geneva.

[12] Thaddeus S, Maine D (1994). Too far to walk: maternal mortality in context. Soc Sci Med.

[13] Pacagnella RC, Cecatti JG, Parpinelli MA, Sousa MH, Haddad SM, Costa ML (2014). Delays in receiving obstetric care and poor maternal outcomes: results from a national multicentre cross-sectional study. BMC Pregnancy Childbirth.

[14] Forshaw J, Raybould S, Lewis E, Muyingo M, Weeks A, Reed K (2016). Exploring the third delay: an audit evaluating obstetric triage at Mulago National Referral Hospital. BMC Pregnancy Childbirth..

[15] Goodman DM, Srofenyoh EK, Olufolabi AJ, Kim SM, Owen MD (2017). The third delay: understanding waiting time for obstetric referrals at a large regional hospital in Ghana. BMC Pregnancy Childbirth..

[16] Knight HE, Self A, Kennedy SH (2013). Why are women dying when they reach hospital on time? A systematic review of the “third delay.”. PLoS One.

[17] Bernard T (2012). Impact analysis of rural electrification projects in sub-Saharan Africa. World Bank Res Obs.

[18] Owili PO, Muga MA, Mendez BR, Chen B (2017). Quality of maternity care and its determinants along the continuum in Kenya: a structural equation modeling analysis. PLoS One.

[19] Apenteng BA, Opoku ST, Ansong D, Akowuah EA, Afriyie-Gyawu E. The effect of power outages on in-facility mortality in healthcare facilities: evidence from Ghana. Glob Public Health. 2016:1–11. 10.1080/17441692.2016.1217031 27533753

[20] Mbonye AK, Asimwe JB. Factors associated with skilled attendance at delivery in Uganda: results from a national health facility survey. Int J Adolesc Med Health. 2010;22:249-56. 10.1515/ijamh.2010.22.2.249 21061925

[21] UNDP (2016). UNDP support to the implementation of the sustainable development goals.

[22] World Bank (2017). Climate-smart healthcare. Low-carbon and resilience strategies for the health sector [internet].

[23] Uganda Bureau of Statistics (2016). 2016 Statistical abstract [internet].

[24] Uganda Bureau of Statistics and ICF International (2018). Uganda demographic and health survey 2016 [internet].

[25] World Bank. World Bank Develoment Indicators. [Internet]. Washington D.C.: World Bank; Available from: http://databank.worldbank.org/data/reports.aspx?source=2&series=SP.DYN.TFRT.IN.

[26] Uganda Bureau of Statistics (UBOS) and ICF Internatinoal Inc (2007). Uganda demographic and health survey 2006 [internet].

[27] Waiswa P, Kallander K, Peterson S, Tomson G, Pariyo GW (2010). Using the three delays model to understand why newborn babies die in eastern Uganda. Trop Med Int Health TM IH.

[28] Ministry of Health, Health Systems 20/20, and Makerere University School of Public Health (2012). Uganda Health System Assessment 2011 [Internet].

[29] Ministry of Health Uganda and Macro International Inc. Uganda service provision assessment survey 2007 [internet]. Kampala, Uganda and Calverton: Ministry of Health and Macro International Inc.; 2008. Available from: https://dhsprogram.com/pubs/pdf/SPA13/SPA13.pdf.

[30] United Nations. Health Facility Energy Needs Assessment Uganda Country Summary Report [Internet]. United Nations Foundation; 2015. Available from: http://poweringhc.org/wp-content/uploads/2018/04/Uganda-Country-Summary-Report-Final-Draft-090115.pdf

[31] Ministry of Health Uganda (2013). Uganda Services Availability and Readiness Assessment 2013 Summary report: key findings in figures [internet].

[32] Institute for Health Metrics and Evaluation (IHME) (2014). Health service provision in Uganda: assessing facility capacity, costs of care, and patient perspectives [internet].

[33] Clinton Health Access Initiative. Data from CHAI Uganda Health Facility Assessment. 2016.

[34] USAID (2013). Maternal and child health integrated program (MCHIP) [internet].

[35] Tripathi V, Stanton C, Strobino D, Bartlett L. Development and validation of an index to measure the quality of facility-based labor and delivery care processes in sub-Saharan Africa. PLoS One. 2015 [cited 2017 Jul 25];10. Available from: http://www.ncbi.nlm.nih.gov/pmc/articles/PMC4479466/10.1371/journal.pone.0129491 PMC4479466 26107655

[36] World Bank. Impact evaluation toolkit for results based financing in health [internet]. World Bank. Available from: https://www.rbfhealth.org/resource/impact-evaluation-toolkit-provides-hands-guidance.

[37] Kruk ME, Larson E, Twum-Danso NAY (2016). Time for a quality revolution in global health. Lancet Glob Health.

[38] Kruk ME, Kujawski S, Mbaruku G, Ramsey K, Moyo W, Freedman LP (2018). Disrespectful and abusive treatment during facility delivery in Tanzania: a facility and community survey. Health Policy Plan.

[39] Koroglu M, Irwin BR, Grépin KA (2019). Effect of power outages on the use of maternal health services: evidence from Maharashtra. India. BMJ Global Health..

