# Supplementary material for: Shedding light on quality of care: a study protocol for a randomized trial evaluating the impact of the Solar Suitcase in rural health facilities on maternal and newborn care quality in Uganda
Source: BMC Pregnancy Childbirth. 2019 Aug 22;19:306. doi: 10.1186/s12884-019-2453-x (PMC6704514; doi:10.1186/s12884-019-2453-x)
Supplement: Supplementary file 1 — Table A1. Additional 17 items composing 37-item quality of maternal care index, as adapted from MCHIP (2013). Table A2. Items composing satisfaction indices. Table A3. Secondary outcomes. (DOCX 19 kb) [file 12884_2019_2453_MOESM1_ESM.docx]

**Additional file 1**

**Table A1. Additional 17 items composing 37-item quality of maternal care index, as adapted from MCHIP (2013)**

| Asks about complications during previous pregnancies. |
| --- |
| Washes his/her hands with soap and water or uses alcohol hand rub before **initial** examination. |
| Takes temperature. |
| Checks fundal height. |
| Checks fetal presentation by palpation of abdomen |
| Checks fetal heart rate with fetoscope/Doppler/ultrasound |
| Performs vaginal examination. |
| Wears sterile surgical gloves for all vaginal examinations. |
| Prepares disposable cord ties/clamps AND sterile scissors/blade. |
| As baby’s head is delivered, supports perineum. |
| Applies traction to the cord while applying suprapubic counter traction. |
| Performs uterine massage immediately following the delivery of the placenta. |
| Checks baby's temperature. |
| Provides assistance to mother with breastfeeding. |
| Sterilizes or uses high-level disinfection for all reusable instruments |
| Disposes of all contaminated waste in leak-proof containers |
| Washes his/her hands with soap and water or uses alcohol hand rub after cleaning up. |

Notes: Each indicator is scored as binary for health worker performed (1) or did not perform (0). Index score for each delivery is fraction of items performed.

**Table A2. Items composing satisfaction indices**

| 1. Satisfaction with light & electricity [binary 0/1 for strongly agree with both]: |
| --- |
| i. I am satisfied with the availability and brightness of light in this facility. |
| ii. I am satisfied with the availability of electricity in this facility. |
|  |
| 2. Overall job satisfaction index [mean score]: |
| i. These days, I feel motivated to work as hard as I can. |
| ii. Overall, I am satisfied with my job. |
| iii. Overall, the morale level at my department is good |
| iv. I plan on staying at this position for the next year. |

Notes: Responses for each item on a 1-5 scale with 1: Strongly Disagree, 2: Disagree, 3: Neutral (Neither Agree nor Disagree), 4: Agree, 5: Strongly Agree.

**Table A3. Secondary outcomes**

| **Outcome** | **Definition** | **Scale of measurement at delivery-level** |
| --- | --- | --- |
| Mean level of brightness over 4 periods of labor and delivery on brightness scale as measured by observer recorded questionnaire | Scale is scored as 1: Very Bright; 2: Somewhat Bright; 3: Dim; 4: Pitch Black | average score over 4 periods |
| Minutes of delivery observation without satisfactory light as measured by observer recorded questionnaire | Satisfactory light source defined in Primary Outcome 2. | number of minutes (continuous) |
| Consistent satisfactory light source as measured by observer recorded questionnaire | Variable equal to 1 if no interruptions of > 2 hours in satisfactory light source during observed deliveries | binary [0/1] |
| Fetal Doppler Use as measured by observer recorded questionnaire | Number of times the provider used a fetal doppler during labor and delivery | number of times (continuous) |
| Phone availability as measured by observer recorded questionnaire | Phone is available and on for deliveries | binary [0/1] |
| APGAR Score Assigned as measured by observer recorded questionnaire | Provider assigns any APGAR score (binary variable equal to 1 if provider assigns an APGAR score (range of 0-10), and equal to 0 if no APGAR score is assigned. | binary [0/1] |
| Basic emergency obstetric care signal functions as measured by facility assessment | Facility reported performance of basic emergency obstetric care signal functions in the past three months. | binary [0/1] |
| Basic emergency newborn care signal functions as measured by facility assessment | Facility reported performance of basic emergency newborn care signal functions in the past three months | binary [0/1] |
| Routine obstetric care signal functions as measured by facility assessment | Facility reported universal practice of routine obstetric care signal functions | binary [0/1] |
| Routine newborn care signal functions as measured by facility | Facility reported universal practice of routine newborn care signal functions | binary [0/1] |
| Time between delivery and when suturing began (minutes) as measured by observer recorded questionnaire |  | number of minutes (continuous) |
| 4-item quality of suturing index as measured by observer recorded questionnaire | Health worker: (1) explains to woman what is happening, (2) cleans area prior to suturing with antiseptic solution, (3) prepares instruments/sutures on a sterile surface, and (4) sutures episiotomy or tears appropriately in layers. | sum of 4 items |
| 10-item patient treatment index as measured by observer recorded questionnaire | Patient treatment/inter-personal quality care index, based on Kruk et al. (2014) | sum of 10 items |
| 14-item Partograph Completeness index as measured by observer recorded questionnaire | Index of variables (from MCHIP (2013) | sum of 14 items |
| Time between contractions as measured by observer recorded questionnaire |  | number of minutes (continuous) |
| Dilation at first exam as measured by observer recorded questionnaire |  | whole number  [0-10] |
| Monthly Delivery Volumes as measured by facility register |  | number of births (continuous) |
| Monthly Nighttime Delivery Volumes as measured by facility register |  | number of births (continuous) |
| Monthly ANC Volumes as measured by facility register |  | number of visits (continuous) |
| Monthly Referral Volumes as measured by facility register |  | number of referrals (continuous) |
| Sensor Brightness as measured by sensor | Recorded brightness at sensor sight in the two hours around delivery of baby | voltage (continuous) |
| Depletion of Solar Suitcase Battery Based on Voltage as measured by sensor | Average difference between solar suitcase load voltage in evening and morning (based on sensor) | voltage (continuous) |
| Depletion of Solar Suitcase Battery Based on Charge Time as measured by sensor | Average time between morning and when the solar suitcase is fully charged, based on sensor | number of minutes (continuous) |
| Solar Suitcase Use for Deliveries as measured by enumerator recorded questionnaire | Enumerator recorded usage of suitcase | binary [0/1] |
| Health Worker Assessment of Overhead Light Availability as measured by health worker survey | Scale is scored as 1. No overhead light, 2. Very poor, 3. Poor, 4. Good, 5. Very good | whole number [1-5] |
| Health Worker Assessment of Task Light Availability as measured by health worker survey | Scale is scored as 1. No overhead light, 2. Very poor, 3. Poor, 4. Good, 5. Very good | whole number [1-5] |
| Health Worker Assessment of Overhead Light Brightness as measured by health worker survey | Scale is scored as 1. No task light, 2. Very poor, 3. Poor, 4. Good, 5. Very good | whole number [1-5] |
| Health Worker Assessment of Task Light Brightness as measured by health worker survey | Scale is scored as 1. No task light, 2. Very poor, 3. Poor, 4. Good, 5. Very good | whole number [1-5] |
| 14-item index of Health worker assessment of impact of blackouts on their ability to perform job functions as measured by health worker survey | Items on scale are scored as 1. Every nighttime delivery, 2. Most nighttime deliveries, 3. Some nighttime deliveries, 4. Few nighttime deliveries, 5. Never | average of 14 items |
| All individual Quality of care items from both the 20-item Tripathi index and the 37-item MCHIP index | Each item as binary variable (37 total items) | binary [0/1] |
| All individual delay items from 6-item delay index | Each item as time in minutes (6 total items) | number of minutes (continuous) |
